# Chinese university students showed less disordered eating during the COVID-19 campus lockdown

**DOI:** 10.1007/s40519-023-01569-w

**Published:** 2023-05-27

**Authors:** Wei Duan, Qiuwei Ding, Sufang Peng, Qing Kang, Lei Guo, Lei Zhang, Yaohui Wei, Zeping Xiao, Juan Fan, Jue Chen

**Affiliations:** 1 Shanghai Mental Health Center, Shanghai Jiao Tong University School of Medicine, Shanghai, 200030 China; 2 The Peddie School, New Jersey, 08520 USA

**Keywords:** COVID-19, Lockdown, Disordered eating, Mental health, University students

## Abstract

**Objective:**

The rapid spread of the Omicron variant of COVID-19 in China had resulted in campus lockdown in many universities since February 2022, profoundly affecting students’ daily lives. Campus lockdown conditions differ considerably from home quarantine, so that the eating patterns of university students may be different. Thus, the current study aimed to: (1) investigate university students’ eating patterns during campus lockdown; (2) identify factors associated with their disordered eating.

**Method:**

An online survey about recent life changes, disordered eating, stress, depression, and anxiety was carried out from April 8th to May 16th, 2022. A total of 2541 responses from 29 provinces/cities of China were received.

**Results:**

2213 participants were included in the main analysis, and other 86 participants were analyzed separately as a subgroup due to their diagnosis of eating disorder. Participants who were undergoing campus lockdown (the lockdown group) showed less disordered eating than those who had never been in campus lockdown (the never-lockdown group), as well as those who had experienced campus lockdown before (the once-lockdown group). However, they perceived more stress and felt more depressed. Being female, higher BMI, gaining weight, increasing exercise, spending more time on social media, higher level of depression and anxiety were all related to disordered eating in the lockdown group.

**Conclusions:**

Disordered eating among Chinese university students was less prevalent during campus lockdown due to the strict and regular diet. However, there is a potential risk of “revenge eating” after campus lockdown ends. Thus, there should be further tracking and related prevention.

**Level of evidence:**

IV, uncontrolled trials without any interventions.

## Introduction

Since February 2022, China had experienced a massive coronavirus outbreak due to the Omicron variant of COVID-19 [1], leading to campus lockdown in many universities to protect students and staff. This has significantly disrupted the lives of students, who were not allowed to leave the campus, with classes and exams being conducted remotely, and face-to-face activities being cancelled or changed to an online style. In some severely affected regions, students were even confined to their dormitories, with the only outdoor activities being nucleic acid PCR testing [2].

Previous studies have demonstrated that while lockdown could effectively halt the spread of the virus, it could also cause many mental health problems, such as stress [3, 4], depression and anxiety [5], insomnia [6] and PTSD [7]. Furthermore, the deterioration of psychological health can lead to problematic eating behaviors during lockdown. Cecchetto et al. [8] found that increased emotional eating and binge eating were related to higher levels of depression, anxiety and perceived stress in Italian people. Robinson et al. [9] showed that most British people experienced difficulties with eating during lockdown due to poor mental health, such as lack of control of eating, more frequent snack consumption and overeating. According to Scarmozzino et al. [10], 46.1% of investigated Italian people took more comfort food (e.g., chocolate, ice-cream, desserts, etc.) during lockdown, attributing this behavior to their heightened anxiety levels.

Meanwhile, university students were particularly vulnerable to those above-mentioned mental health problems during lockdown [11]. And they were the highly risky population of eating disorders [12], due to the common body dissatisfaction in this population, and stressors such as academic stress, and peer pressure [13, 14], etc. Thus, it is of great importance to pay attention to their psychological health and eating patterns during lockdown and implement specific interventions if necessary.

Previous studies on Chinese university students during lockdown were mostly based on their home quarantine and focused on overall mental health states, such as stress [11, 15, 16], depression [4, 11, 16–18], anxiety [4, 11, 16, 17, 19, 20] and PTSD [19]. However, little was known about their psychological states during campus lockdown and how it would impact their eating patterns. Living conditions of campus lockdown were significantly different from home quarantine. For instance, the living space was more limited as most students were confined in dormitories, far smaller than their homes. In addition, meals were prepared and delivered to students on time by schools, so there was no need for students to worry about their meals. But they were given no choice of other food that they preferred because of limited food supply.

Thus, the present study aimed to investigate whether the campus lockdown influenced the mental health and eating patterns of Chinese university students, as well as the related factors to disordered eating. There would be three subgroups to be studied, based on students’ lockdown history: the never-lockdown group that had never experienced any campus lockdown, the lockdown group that was undergoing campus lockdown, as well as the once-lockdown group that had finished their campus lockdown already. We hypothesized that the lockdown group would experience worse mental health than the other two groups, and thus more severe disordered eating would show up. Additionally, we expected that the related factors to disordered eating of the lockdown group would be being younger and female, higher BMI, longer lockdown duration, gaining weight, decreasing exercises, difficult food access and more time spent on social media.

## Methods

### Research design and participants

This cross-sectional study applied an online survey to investigate disordered eating and its related factors in Chinese university students during the campus lockdown caused by the Omicron variant of COVID-19. We collected online data via a Chinese survey website Wenjuanxing (www.wjx.com) from April 8th to May 16th, 2022, targeting on Chinese university undergraduate students. The survey link was also shared by social media (e.g., WeChat moments, WeChat groups, etc.) to obtain as a large sample as possible. Participants who finished the survey and passed the attention check items would have a 50% chance of receiving Rmb5 yuan payments. This study was approved by the Ethics Committee of the Shanghai Mental Health Center and the Institutional Review Board of Shanghai Mental Health Center (2020–2032). The confidentiality of responses was assured to all the participants, and their electronic informed consents were obtained online.

A total of 2541 responses were obtained, and 2299 participants from 29 provinces/cities of China were finally included in the statistical analysis. We excluded 154 participants who spent less than 3 min on the survey, 36 with poor data quality (such as implausible weight or height data or failed quality checks), and 52 participants who were not in campus lockdown, but rather quarantined in hospital/hotel or at home. Detailed distribution of the sample in each province or city of China was presented in supplementary materials (S1).

According to the participants’ lockdown history, they were divided into three groups: the lockdown group, the never-lockdown group, and the once-lockdown group. The first group were still undergoing campus lockdown when participating in the online survey, while the second group had never experienced campus lockdown, and the third group had previously experienced campus lockdown before taking part in the survey.

### Measures

#### Demographics

Age, gender, height, weight, and region were investigated. Body Mass Index (BMI) was calculated by self-reported weight and height: weight/height^2^ (kg/m^2^) and categorized into underweight (< 18.5 kg/m^2^), normal (18.5–23.9 kg/m^2^), overweight (24–27.9 kg/m^2^), and obese (≥ 28 kg/m^2^) [21].

#### Life changes

The following questions were asked regarding the life changes. For the lockdown group, it was life changes during campus lockdown compared to before. And it was about life changes in the past month versus before if participants were not in campus lockdown. First, lockdown experiences were measured by whether they had been in quarantine since 2022 and currently. Second, quarantine format and duration were asked only for the currently quarantined group. Other questions were answered by all participants, including changes of weight, food access, and exercise. Besides, the time spent on social media per day was also asked to investigate the media influence. All these questions are presented in the supplementary materials (S2).

#### Disordered eating

Participants completed the Eating Disorder Examination Questionnaire, 6th version (EDE-Q 6.0), which has been well validated in China [22]. The cutoff score of EDE-Q 6.0 is 1.27. Besides, item 13–18 indicates problematic eating behaviors such as binge eating, losing control of eating, self-induced vomiting, excessive exercise, etc. The Cronbach’s alpha coefficients of the whole EDE-Q and the four subscales were as follow: the whole questionnaire (α = 0.90), Restraint Eating subscale (α = 0.89), Eating Concern subscale (α = 0.85), Weight Concern subscale (α = 0.85), and Shape Concern subscale (α = 0.90).

#### Perceived stress

The Chinese version of the Perceived Stress Scale 10-item version (PSS-10; [23]) was applied to measure the stress. Each item was rated on a 5-point Likert scale (0 for never, 5 for always). A higher total score represented a higher perceived stress level. The Cronbach’s alpha coefficient of the PSS-10 in the present study was 0.88.

#### Negative emotions

The Generalized Anxiety Disorder Assessment 7-item version (GAD-7; [24]) and the Patient Health Questionnaire 9-item version (PHQ-9; [25]) were used to assess the anxiety and depression of participants, respectively. Both have been validated in the Chinese version with good reliabilities and validities [26, 27]. The same response options of these two questionnaires are the same, which were 0 for “not at all,” 1 for “several days,” 2 for “more than half the days,” and 3 for “nearly every day.” They use the same cut-off score as 10. The Cronbach’s alpha coefficient of GAD-7 in this study was 0.94, and the PHQ-9 was 0.91.

## Analysis

The following analyses were conducted in SPSS (version 25.0) at an alpha level of 0.05. The only figure in this thesis was produced in R (version 4.2.0) with the package ggplot2 [28]. There were no missing data as the forced-choice/fill design was applied in the online survey. The Shapiro–Wilk test was used to examine the normality of data distribution, which showed that all variables of interest were against the normality distribution. Therefore, non-parametric tests were used for further analyses. A descriptive analysis was done using the median and interquartile range (*IQR*) for continuous measures, as well as the counts (*n*) and percentages (%) for categorical variables. Then Kruskal–Wallis tests were used to test the differences of age, time spent on social media, disordered eating and mental health (i.e., stress, depression and anxiety) among the three groups. Chi-square tests were used to compare the categorical variables. Post-hoc comparisons were done with Bonferroni correction if any group differences were detected, and the adjusted *p* values were reported and compared with 0.05. Finally, to explore the related factors to the disordered eating of the lockdown group from different levels, we conducted a multiple hierarchical regression analysis. In step 1 of the model, demographic variables (age, gender, BMI) were put into. In step 2, several potential variables about campus lockdown were entered, such as lockdown duration, changes in life (exercise increase/decrease, weight gain/loss, harder/easier food access), and time spent on social media. Finally, measures of mental health were put into the model in step 3, i.e., perceived stress, anxiety, and depression.

## Results

### Overall sample descriptions

The total sample comprised 2299 Chinese university students. 3.74% of them (*n* = 86) reported a current or past diagnosis of an eating disorder, which were removed from the main analysis and analyzed further as subgroups separately (see S3) as this study mainly focus on the general population.

Thus, there were 2213 participants included in main analysis, predominantly female (*n* = 1452, 65.61%). Their age ranged from 18 to 25 years old, with a median of 20 years old (IQR = 2). The median of BMI was 20.57 kg/m^2^ (IQR = 3.70). Most of the participants were with a normal BMI (*n* = 1460, 65.97%). 412 participants (18.61%) were underweight. 12.20% (*n* = 270) were overweight, and 3.21% (*n* = 71) were obese.

### Demographic characteristics and life changes

Students in these three groups were at the same age (see Table 1; *H* = 0.502, *p* = 0.778), of which the median was 20 years old (IQR = 2). Same as the overall sample, most of the participants in these three groups were females (*χ*^2^ = 5.653, *p* = 0.098). There were no significant group differences as for BMI (*H* = 2.816, *p* = 0.245) or its categories (*χ*^2^ = 7.541, *p* = 0.274). Most participants (63.99–68.37%) in all three groups were with a normal BMI.

**Table 1:** Comparisons of demographics and living conditions

	Overall (*n* = 2213) Median (IQR)/*n* (%)	Never-lockdown group (*n* = 781) Median (IQR)/*n* (%)	Lockdown group (*n* = 971) Median (IQR)/*n* (%)	Once-lockdown group (*n* = 461) Median (IQR)/*n* (%)	*H*/*χ*^2^
Age	20 (2)	20 (2)	20 (2)	20 (2)	0.502
Gender					5.653
Male	761 (34.38%)	283 (36.23%)	310 (31.92%)	168 (36.44%)	
Female	1452 (65.61%)	498 (63.76%)	661 (68.07%)	293 (63.55%)	
BMI (kg/m^2^)	20.57 (3.70)	20.57 (3.53)	20.42 (3.73)	20.81 (3.98)	2.816
BMI category					7.541
Normal	1460 (65.97%)	534 (68.37%)	631 (64.98%)	295 (63.99%)	
Underweight	412 (18.61%)	136 (17.41%)	195 (20.08%)	81 (17.57%)	
Overweight	270 (12.20%)	86 (11.01%)	114 (11.74%)	70 (15.18%)	
Obese	71 (3.21%)	25 (3.20%)	31 (3.19%)	15 (3.25%)	
Exercise changes					160.268***
The same as before	476 (21.50%)	215 (27.52%)	148 (15.24%)	113 (24.51%)	
Increased	636 (28.73%)	272 (34.82%)	193 (19.87%)	171 (37.09%)	
Decreased	1101 (49.75%)	294 (37.64%)	630 (64.88%)	177 (38.39%)	
Weight changes					20.366***
The same as before	1442 (65.16%)	549 (70.29%)	591 (60.86%)	302 (65.50%)	
Weight gain	379 (17.12%)	116 (14.85%)	176 (18.12%)	87 (18.87%)	
Weight loss	392 (17.71%)	116 (14.85%)	204 (21.00%)	72 (15.61%)	
Changes of food access					312.360***
The same as before	1017 (45.95%)	487 (62.35%)	249 (25.64%)	281 (60.95%)	
Harder	1064 (48.07%)	246 (31.49%)	669 (68.89%)	149 (32.32%)	
Easier	132 (5.96%)	48 (6.14%)	53 (5.45%)	31 (6.72%)	
Social media use (hours per day)	5 (5)	5 (5)	6 (5)	4 (4)	46.234***

***p < 0.001

During the campus lockdown, some changes happened to participants’ exercise, weight and access to food. There were more participants in the lockdown group felt themselves exercising less (64.88%) and losing weight (21.00%), more than other two groups (exercise changes: *χ*^2^ = 160.268, *p* < 0.001; weight changes: *χ*^2^ = 20.366, *p* < 0.001). And 68.89% participants found it harder to get food during campus lockdown, while other two groups mostly felt the food access remaining the same as before (*χ*^2^ = 312.360, *p* < 0.001). Moreover, participants in the lockdown group spent more time on social media (*H* = 46.234, *p* < 0.001) than the other two groups.

### Disordered eating and mental health state

**Fig. 1:**
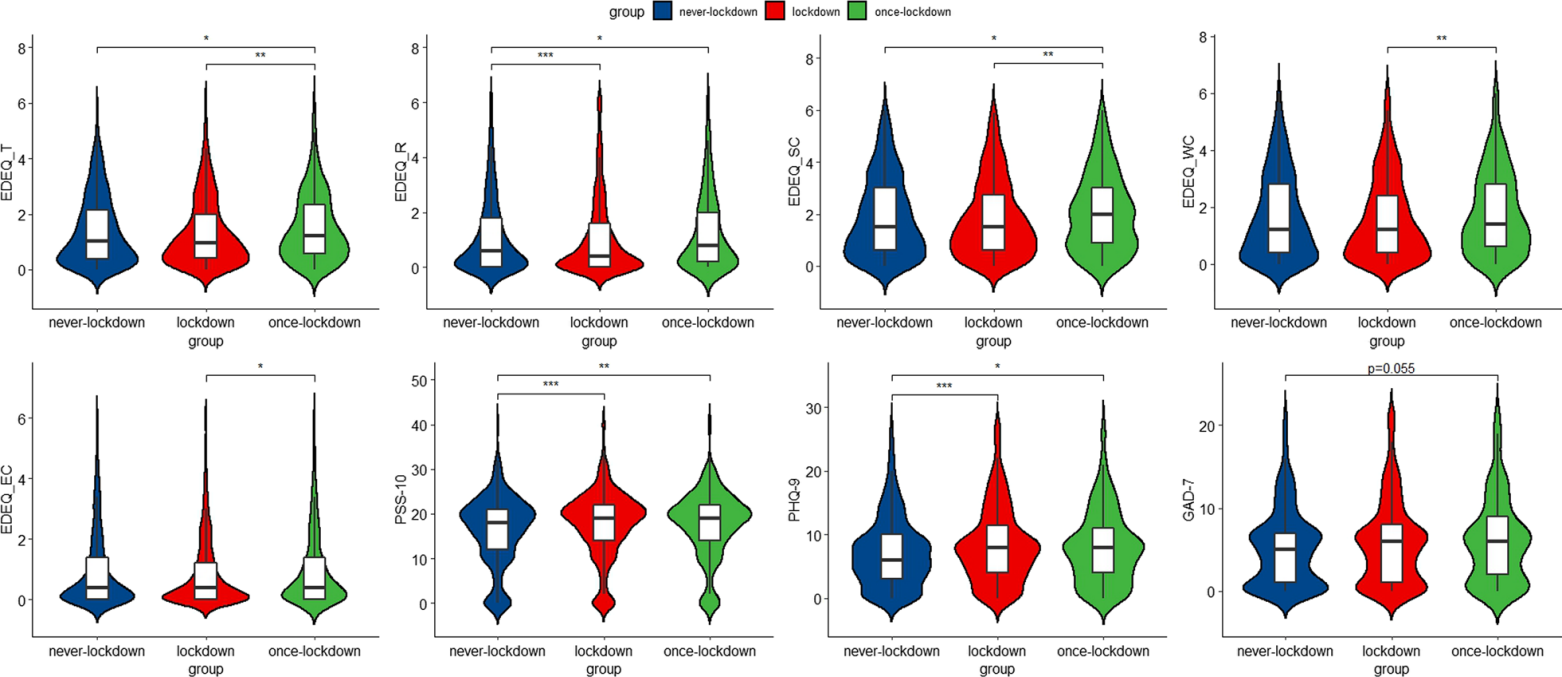
Comparisons of the EDE-Q total scores and its three subscales, as well as the PSS, PHQ and GAD. *p < 0.05, **p < 0.01, ***p < 0.001. *EDEQ_T* T Total scores of the Eating Disorder Examination Questionnaire (EDE-Q), *EDEQ*_R Scores of restraint eating subscale of EDE-Q, *EDEQ_SC* scores of shape concern subscale of EDE-Q, *EDEQ_WC* scores of weight concern subscale of EDE-Q, *EDEQ_EC* scores of eating concern subscale of EDE-Q, *PSS-10* Perceived Stress Scale-10, *PHQ-9* Patient Health Questionnaire-9, *GAD-7* Generalized Anxiety Disorder-7

Table 2 shows detailed information on disordered eating and mental health states, with group differences shown in Fig. 1. Over half of the participants (*n* = 1269, 57.34%) in the overall sample showed severe eating problems, as their EDE-Q total scores were over 1.27. There were significant group differences in the EDE-Q total scores as well as its four subscales among the three groups. Post-hoc comparisons confirmed that the lockdown group was significantly lower than the once-lockdown group for all of the scores (Fig. 1). Besides, once-lockdown group also got higher scores in Restraint Eating subscale (*p* = 0.027) and Shape Concern subscale (*p* = 0.034) than the never-lockdown group. What’s more, The lockdown group also had fewer participants (*n* = 336, 38.80%) at risk of eating disorders than the once-lockdown group (*χ*^2^ = 12.707, *p* = 0.002).

**Table 2:** Comparison of disordered eating, perceived stress, and negative emotions between the lockdown group and the never-lockdown group

	Overall (*n* = 2213) Median (IQR)/*n* (%)	Never-lockdown group (*n* = 781) Median (IQR)/*n* (%)	Lockdown group (*n* = 971) Median (IQR)/*n* (%)	Once-lockdown group (*n* = 461) Median (IQR)/*n* (%)	*H*/*χ*^2^
EDEQ					
Total scores	1.01 (1.66)	1 (1.76)	0.96 (1.60)	1.23 (1.76)	13.094**
Cutoff score					12.707**
< 1.27	1269 (57.34%)	435 (55.69%)	595 (61.27%)	239 (51.84%)	
≥ 1.27	944 (42.65%)	346 (44.3%)	376 (38.72%)	222 (48.15%)	
Restraint eating	0.60 (1.80)	0.60 (1.80)	0.40 (1.60)	0.80 (1.80)	16.505***
Eating concern	0.40 (1.40)	0.40 (1.40)	0.40 (1.20)	0.40 (1.40)	6.377*
Weight concern	1.20 (2.20)	1.20 (2.40)	1.20 (2.00)	1.40 (2.20)	9.766**
Shape concern	1.50 (2.25)	1.50 (2.38)	1.50 (2.13)	2.00 (2.19)	10.472**
Binge eating, n (%)	1035 (46.83%)	393 (50.44%)	408 (42.06%)	234 (50.75%)	15.813***
Lost control when eating, *n *(%)	824 (37.31%)	311 (39.82%)	333 (34.4%)	180 (39.21%)	6.320*
Vomiting, *n *(%)	140 (6.32%)	59 (7.55%)	54 (5.56%)	27 (5.85%)	3.118
Laxative misuse, *n *(%)	128 (5.78%)	55 (7.04%)	50 (5.14%)	23 (4.98%)	3.521
Excessive exercise, *n *(%)	820 (37.18%)	293 (37.7%)	345 (35.64%)	182 (39.56%)	2.196
PSS-10	19.00 (9.00)	18.00 (9.00)	19.00 (8.00)	19.00 (8.00)	17.538***
PHQ-9					
Total scores	7.00 (6.00)	6.00 (7.00)	8.00 (8.00)	8.00 (7.25)	16.018***
Cutoff score					9.612**
< 10	1542 (69.67%)	576 (73.75%)	652 (67.14%)	314 (68.11%)	
≥ 10	671 (30.32%)	205 (26.24%)	319 (32.85%)	147 (31.88%)	
GAD-7					
Total scores	5.00 (6.00)	5.00 (6)	6.00 (7)	6.00 (7)	6.730*
Cutoff score					5.923*
< 10	1794 (81.06%)	653 (83.61%)	780 (80.32%)	361 (78.3%)	
≥ 10	419 (18.93%)	128 (16.38%)	191 (19.67%)	100 (21.69%)	

*EDEQ* Eating Disorder Examination Questionnaire, *PHQ-9* Patient Health Questionnaire-9, *GAD-7* Generalized Anxiety Disorder-7, *PSS-10* Perceived Stress Scale-10

*p < 0.05, **p < 0.01, ***p < 0.001

Regarding specific disordered eating behaviors, 46.83% of participants (*n* = 1035) reported binge eating during campus lockdown or in the past 28 days. There were fewer participants (*n* = 408, 42.05%) in the lockdown group experienced binge eating than the other two groups (*χ*^2^ = 15.813, *p* < 0.001). 37.31% of participants (*n* = 824) experienced losing control during eating, in which the lockdown group was slightly less than the other two groups (*χ*^2^ = 6.320, *p* = 0.042). Moreover, the three groups were similar as to the proportions of vomiting, laxative misuse and excessive exercise (*p* > 0.05). In the overall sample, 6.32% had vomited (*n* = 140), 6.11% had misused laxatives (*n* = 120) and 37.00% had exercised excessively (*n* = 727).

Overall, approximately 30.32% of participants (*n* = 671) felt depressed, and 18.93% of participants (*n* = 419) felt anxious. The never-lockdown group felt less stressed and depressed than both the lockdown group (stress and depression: *p* < 0.001) and the once-lockdown group (stress: *p* = 0.005; depression: *p* = 0.027). As to the anxiety, post-hoc comparisons showed that the never-lockdown group was slightly less anxious than the once-lockdown group (*p* = 0.052), with also slightly less participants scoring more than 10 in GAD-7 than other two groups (*χ*^2^ = 5.923, *p* = 0.052).

### Related factors to disordered eating in the lockdown group

We did not detect any multicollinearity among the regression model as the variance inflation factors (VIF) for them were between 1.002 and 3.242.

Results of the three models from the regression analysis were shown in Table 3. The first model had an adjusted R^2^ of 0.124, suggesting that demographic variables such as age, gender and BMI could account for 12.4% of the variance in disordered eating during campus lockdown. However, age was not a significant predictor (*p* = 0.628). Females and those with higher BMI were more likely to experience disordered eating during campus lockdown (*p* < 0.001). Model 2 significantly improved upon model 1 (*F change* (8, 943) = 0.053, *p* < 0.001), with the adjusted R^2^ increasing from 0.124 to 0.180. In this block, lockdown duration (*p* = 0.546) was not significant. Participants who reported weight gain, increased exercise, and difficulty in accessing food during campus lockdown were at higher risk for disordered eating. And model 3 improved further, *F change* (3, 940) = 54.064, *p* < 0.001, and the adjusted R^2^ increased from 0.180 to 0.298. Mental health variables were significant except for perceived stress (*p* = 0.418). The more depressed or anxious participants felt during campus lockdown, the more likely they would be to have eating problems.

**Table 3:** Multiple hierarchical regression analysis of related factors to disordered eating in the lockdown group

	Model 1		Model 2		Model 3	
	B	*p*	B	*p*	B	*p*
Age	− 0.015	0.628	− 0.040	0.183	− 0.053	0.056
Gender^a^	**0.166**	** < 0.001**	**0.140**	** < 0.001**	**0.141**	** < 0.001**
BMI	**0.367**	** < 0.001**	**0.361**	** < 0.001**	**0.356**	** < 0.001**
Lockdown duration			0.018	0.546	− 0.009	0.740
Weight gain vs. the same as before			**0.145**	** < 0.001**	**0.106**	** < 0.001**
Weight loss vs. the same as before			0.047	0.130	0.037	0.193
Increased exercise vs. the same as before			**0.099**	**0.014**	**0.094**	**0.012**
Decreased exercise vs. the same as before			− 0.015	0.704	− 0.020	0.586
Harder food access vs. the same as before			**0.110**	**0.001**	0.038	0.216
Easier food access vs. the same as before			0.040	0.280	0.009	0.756
Time spent on social media			**0.156**	** < 0.001**	**0.099**	** < 0.001**
Perceived stress					0.030	0.418
Depression					**0.158**	**0.001**
Anxiety					**0.199**	** < 0.001**

Bold: significantly related factors. *p < 0.05, **p < 0.01, ***p < 0.001

Codes for categorical independent variables: ^a^male = 0, female = 1. Model 1: *F* (3, 860) = 30.642, *p* < 0.001, Adj. R^2^ = 0.093. Model 2: *F change* (8, 852) = 4.800, *p* < 0.001, Adj. R^2^ = 0.124. Model 3: *F change* (3, 849) = 61.368, *p* < 0.001, Adj. R^2^ = 0.278

## Discussion

The present study explored the eating patterns and related factors of Chinese university students during campus lockdown. We found that students in campus lockdown showed lower EDE-Q total scores, indicating less disordered eating compared to those who were not in campus lockdown, especially those participants who had once experienced lockdown before. They had less restraint eating, and were less concerned about eating, their weight and shape (Table 2). Moreover, as to the disorder eating behaviors, less participants in the lockdown group reported binge eating and losing control of eating. But the compensatory behaviors, such as vomiting, misusing laxative, and exercising excessively, were similar among the three groups.

These findings were contrary to our hypotheses and counterintuitive, as previous studies indicated that people during lockdown were more likely to show disordered eating behaviors, such as restricted dietary [29], emotional eating and binge eating [8], because of their negative feelings during lockdown. There might be some clues in the differences between campus lockdown and usual lockdowns. During campus lockdown, regular meals were well-prepared and delivered centrally, three times a day. Delivery food and express were not accessible, as the Omicron was raging and delivery was forbidden by universities to cut down the potential spread from outside. Therefore, students were given no choice of food due to the limited food supply during campus lockdown. It was also shown in our study that, more participants in the lockdown group mostly found it hard to get food than the other two groups (Table 1). In such circumstances, it was possible that healthy and regular eating patterns may have developed, leading to significantly fewer disordered eating cognitions and binge eating. After all, there were no enough food for participants to binge during campus lockdown. Finally, other compensatory behaviors were of no group differences, maybe because participants who conducted these kinds of behaviors were already at risk of eating disorder, and thus show the similar patterns with eating disorder subgroup (S3). It indicated the limited role of regular diet during campus lockdown in the prevention of disordered eating.

Moreover, it is notable that the once-lockdown group was significantly more severe in terms of disordered eating than the lockdown group. This implies that students in the once-lockdown group would “relapse” after campus lockdown. There may be a disinhibition effect that would worsen participants’ disordered eating when the lockdown ended. Once all restrictions ended, university students would have access to all kinds of food, such as fast food, snacks, etc., which they could use to compensate for the earlier limitations. Future follow-up studies are needed to track the long-term effect of campus lockdown on students’ eating patterns.

However, there were no significant group differences among the eating disorder sample (S3). This may be because the highly-restricted situation in campus lockdown only buffered the deterioration of eating disorder symptomatology to some extent for eating disorder patients. Besides, they was actually behaved better during campus lockdown if we compared them with those EDs who reported increased disordered eating during usual lockdown [30–32].

As to the related factors about disordered eating during campus lockdown, the multiple hierarchical regression analysis showed that being female and having a higher BMI were correlated with disordered eating, which is consistent with previous studies [33–36]. During campus lockdown, participants who gained weight and increased exercise were more likely to display disordered eating. It is understandable when considering the whole lockdown group. Most participants in the lockdown group (64.88%) experienced decreased exercise due to quarantine in dormitories or campus. If participants were still able to exercise more than usual when they were quarantined in campus or even dormitories, they might have been strongly motivated by their disordered eating cognitions. Similarly, there were more students (21.00%) reported weight lost during campus lockdown than the other two groups (never-lockdown: 13.53%, once-lockdown: 14.86%). So participants who found themselves gaining weight would be more worried about their weight and thus performed disordered eating. Lastly, we found that depression and anxiety could positively predict disordered eating in the lockdown group. This was consistent with many previous studies from different countries, showing that poor mental health due to lockdown would lead to unhealthy and even dysfunctional eating [37–39].

All in all, our study prompted that regular diet during lockdown could be helpful for prevention of disordered eating. It is easy for people to feel messed and stressed in lockdown. Healthy lifestyle plays an important role in life reorganization during lockdown [40], such as regular and healthy diet that implemented in our study, as well as enough physical activity [2, 34, 41], etc. Therefore, basic order in life can be built up to resist mental health crisis during lockdown.

### What is already known on this subject?

People are at risk of poor mental health during lockdown, such as stress, depression and anxiety. Eating could be a way of emotion regulation, so people are more likely than usual to show disordered eating during lockdown, such as emotional eating, binge eating, etc.

### What does this study add?

During campus lockdown, when students were following a strict diet, they were less likely to experience disordered eating. It suggests that a strict diet during lockdown could prevent disordered eating to some extent. But there might be a rebound effect after the temporarily special time is over, which needs constant attention and related prevention.

## Conclusions

This study found that Chinese university students who were in campus lockdown exhibited significantly less disordered eating than those who were not. This finding may be attributed to the strict diet enforced during lockdown. However, poorer mental health states due to lockdown could still predict the severity of disordered eating. Thus, our study highlights the importance of maintaining a regular and healthy diet during times of stress, such as lockdowns. Furthermore, universities should prioritize the mental health of their students during campus lockdown. And continuous tracking is needed for the long-term influence of campus lockdown on students’ eating patterns. Targeted intervention should be implemented on time if needed.

## Limitation

First, the cross-sectional survey here could not give any causation that the decreased disordered eating presented in our study was exclusively the result of campus lockdown. Second, although the online survey is convenient in the current lockdown situation, there is also the problem of data quality. We tried to minimize this problem in the data analysis. For example, we deleted all the answers that were finished in less than 3 minutes. However, there might be other undiscovered potential impacts due to online data collection. Besides, there may be self-report bias in the survey. Third, this study shed light on some changes that happened to Chinese university students’ life during lockdown, but the measures were still crude and not all-around enough. There were probably some other potential factors related to disordered eating during campus lockdown. For example, residing in cramped dormitories with multiple roommates is likely to lead to interpersonal conflicts, which might induce disordered eating [42]. Finally, it is worth noting that our sample was drawn from 29 provinces and cities across China (S1), and the different lockdown measures implemented across regions may have influenced the outcomes of the study.

## Data Availability

The data used in this study are available from the corresponding author.
